# The paradigm shift in treatment of severe venous thromboembolism

**DOI:** 10.1590/1677-5449.202300952

**Published:** 2024-03-04

**Authors:** Fábio Henrique Rossi, Francisco José Osse, Patricia Ellen Thorpe

**Affiliations:** 1 Instituto de Excelência em Doenças Venosas - IEDV, São Paulo, SP, Brasil.

**Keywords:** venous thromboembolism, deep vein thrombosis, pulmonary embolism, multidisciplinary, thrombectomy, mechanical thrombolysis

## Abstract

Pulmonary embolism (PE) is the third leading cause of cardiovascular death and the main cause of preventable in-hospital death in the world. The PERT® (Pulmonary Embolism Response Team) concept involves multidisciplinary diagnosis and immediate treatment. Deep venous thrombosis (DVT) is the initial cause of most cases of PE and is responsible for complications such as chronic thromboembolic recurrence, postthrombotic syndrome, and chronic thromboembolic pulmonary hypertension. An aggressive approach to severe cases of iliofemoral DVT similar to the PERT® system can not only reduce the immediate risk of PE and death but can also reduce later sequelae. New percutaneous techniques and mechanical thrombectomy devices for venous thromboembolism (VTE) have shown encouraging clinical results. We propose the development of an expanded concept of rapid response to VTE, which involves not only PE (PERT®) but also severe cases of DVT: the Venous Thromboembolism Response Team (VTERT®).

## INTRODUCTION

Chronic venous disease (CVD) has the highest prevalence of all vascular conditions and can severely compromise quality of life, requiring long-term medical care, at high personal and social cost. Venous thromboembolism (VTE) is related to deep venous thrombosis (DVT) and/or pulmonary embolism (PE) and to the obstructive venous syndromes that can cause immediate risk and late sequelae, the most prevalent of which is postthrombotic syndrome (PTS), which affects from 30 to 50% of people diagnosed with proximal iliofemoral DVT.^1^ Despite great advances in anticoagulant drugs that are ever safer and more effective, rates of chronic thromboembolic complications have remained unchanged, clearly demonstrating that there is room to seek improvements in therapeutic management.

Pulmonary embolism is the third most common cause of cardiovascular death, behind only acute myocardial infarction (AMI) and stroke, and is the principal cause of avoidable in-hospital deaths.^1^ The abdominal-pelvic venous obstructive syndromes (Cockett/May-Thurner Syndrome, Nutcracker syndrome, and pelvic venous disease) have been associated with occurrence of acute VTE and advanced CVD and can also provoke incapacitating signs and symptoms, such as lumbar pain and hematuria, chronic pelvic pain, dyspareunia, and varicocele. Although DVTs have a predilection for the lower limbs, growing use of peripherally inserted central catheters (PICCs), pacemakers, and hemodialysis, etc. has increased the incidence of DVT and of central venous obstruction, associated with reduced survival of chronic kidney disease patients on hemodialysis.^2^

The incidence of VTE in the general population varies from 60 to 165 new cases per 100,000 people per year and DVT is its most common presentation (85% of all cases). When the incidence of DVT, PE, or both events combined is analyzed in respect of age, there is an exponential increase from the fifth/sixth decade of life onwards, and other risk factors that are common in Western populations, such as obesity, inactivity, smoking, and hormone treatments can also increase incidence.^3^

Deep venous thrombosis is classically divided into two phases: acute, during which the thrombus has the histological composition of a clot, with multiple fibrin fibers trapping red blood cells, platelets, and plasma, and which also provoke adhesion of the clot to the venous wall; and the late phase, when intrinsic and extrinsic reactions in response to formation and presence of the thrombus act to dissolve it, but when there is also fibrotic organization (phlebosclerosis) which, in turn, is responsible for obstruction and entrapment of venous flow and, ultimately, for PTS. During the acute phase, clot fragments may be dislodged into the pulmonary circulation, triggering the most serious of its complications, PE, which can cause death, depending on the size of the clot. Among survivors, the obstructive sequelae provoked by phlebosclerosis that occur during the chronic phase can provoke obstruction of abdominal and pelvic, which in turn veins are risk factors for de novo acute events and PE relapse.^2,3^

Data from a large insurance company in the United States revealed that they received 5.2 claims per 1,000 insured people and that severe CVD was responsible for half of these. It was found that 8.6% of the United States population (mean age: 46.3 years) have a clinically relevant venous disease and that the problem is severe in 3% of these individuals, requiring medical treatment or hospital admission.^3^ Varicose ulcers affect an average of 1.5 to 2 million individuals annually in the United States, at an average annual cost of U$ 850, not including hospital costs, lost work days, or medical products.^3^ As such, recognizing that venous disorders are truly a public health issue is the first step towards development of a responsible management policy, through which effective interventions can be implemented before venous disease becomes chronic, incapacitating, and costly.^4^

We know that PE is a serious disease and is associated with a high risk of mortality which, in turn, can be reduced with early diagnosis and treatment. However, diagnosis is not always considered if acute chest pain, cardiopulmonary collapse, and hemodynamic instability are present. It is not uncommon with these patients, who are treated by the emergency department or intensive care specialists, who are responsible for diagnosis and initial treatment decisions, that PE is not considered in the differential diagnosis of acute cardiopulmonary syndromes. In the majority of cases, the interventional specialist is only called later, to already serious cases, very often when the patient is already being treated with systemic infusion of thrombolytic agents, presenting hemorrhage, hemodynamic instability, and cardiogenic shock, which are clinical situations that are difficult to reverse. The development of percutaneous treatment techniques, primarily mechanical thrombectomy, together with their current encouraging clinical results, and also differences in the levels of experience and learning curves of interventional teams, cause a lack of uniformity and increase the complexity of therapeutic decision making and management of serious PE cases. This has encouraged the development of multidisciplinary rapid response teams to deal with PE (known as pulmonary embolism response teams - PERT®), which include professionals from a range of different specialties and are simultaneously involved in the processes of diagnosis, assessment, and treatment, primarily of those patients identified as being at high risk of death.^5^

The PERT® concept extends to improvement of care and therapeutic management of PE cases, with the basic objective of preventing avoidable deaths from cardiopulmonary complications, promoting immediate implementation of treatment, through building consensus between different specialties after individualized assessment of cases identified in hospital. It is intended to improve organization and utilization of the institution’s resources, with each team member and specialty contributing their specific knowledge to format and standardize management of severe PE cases. In consequence of these actions, it has been observed that there is a gradual, but sustained, increase in diffusion of general and specific knowledge about use of advanced treatments, integrating the several different specialties around a shared objective, resulting not only in increased efficacy and safety, but also in cost reductions.^6^ The concept of PERT® and its accompanying flow diagrams were pioneered at the Massachusetts General Hospital (Harvard Medical School) and reviews of the results of many of the different PERT® services that have been set up have been demonstrating reductions in morbidity and mortality, in the mean time spent in hospital, and in overall treatment costs.^7-9^ If we recognize the clinical necessity and the promising results obtained by “immediate and anticipated intervention to avoid complications” in PE patients with hemodynamic instability, in addition to indications for intervention in serious cases of acute proximal DVT, it seems reasonable, as vascular surgeons and specialists in invasive treatment of VTE, to apply the same concepts embodied in PERT®, not only to PE patients, but also to cases of severe DVT. By so doing, we will not only be relieving the acute symptoms, but also preventing PE, its recurrence, and its chronic complications.^10^

Considering these concepts, we believe that there is an urgent need for a multidisciplinary diagnostic and therapeutic approach to severe DVT cases and so we propose a paradigm shift in the clinical management of severe cases involving the iliac-femoral venous axis, in line with what is happening with respect to PE. Research Ethics Committee approval was waived by our institution.

## CONCEPT: VTE + PERT® = VTERT®

The VTERT® concept is based on a multidisciplinary approach not only to PE cases, but also to those cases of severe proximal DV, in which there is elevated risk of PTS and PE, in patients admitted by the several different departments of a given hospital. The primary objective is to increase the speed of identification, assessment, diagnosis, and treatment of these patients through a pre-established risk stratification process, working along the same lines as other clinical rapid response teams (AMI, stroke, and PE), during the acute phase, as widely implemented in many hospitals in many countries.^11^

The immediate response to requests for assessment of these cases is followed by discussion within the pre-established multidisciplinary team to arrive at the correct decision on what treatment to be initiated, targeting not only the immediate clinical result, with improvement of symptoms and reduction of the immediate risk of death, but also prevention of future complications and relapses.

**Figure 1:**
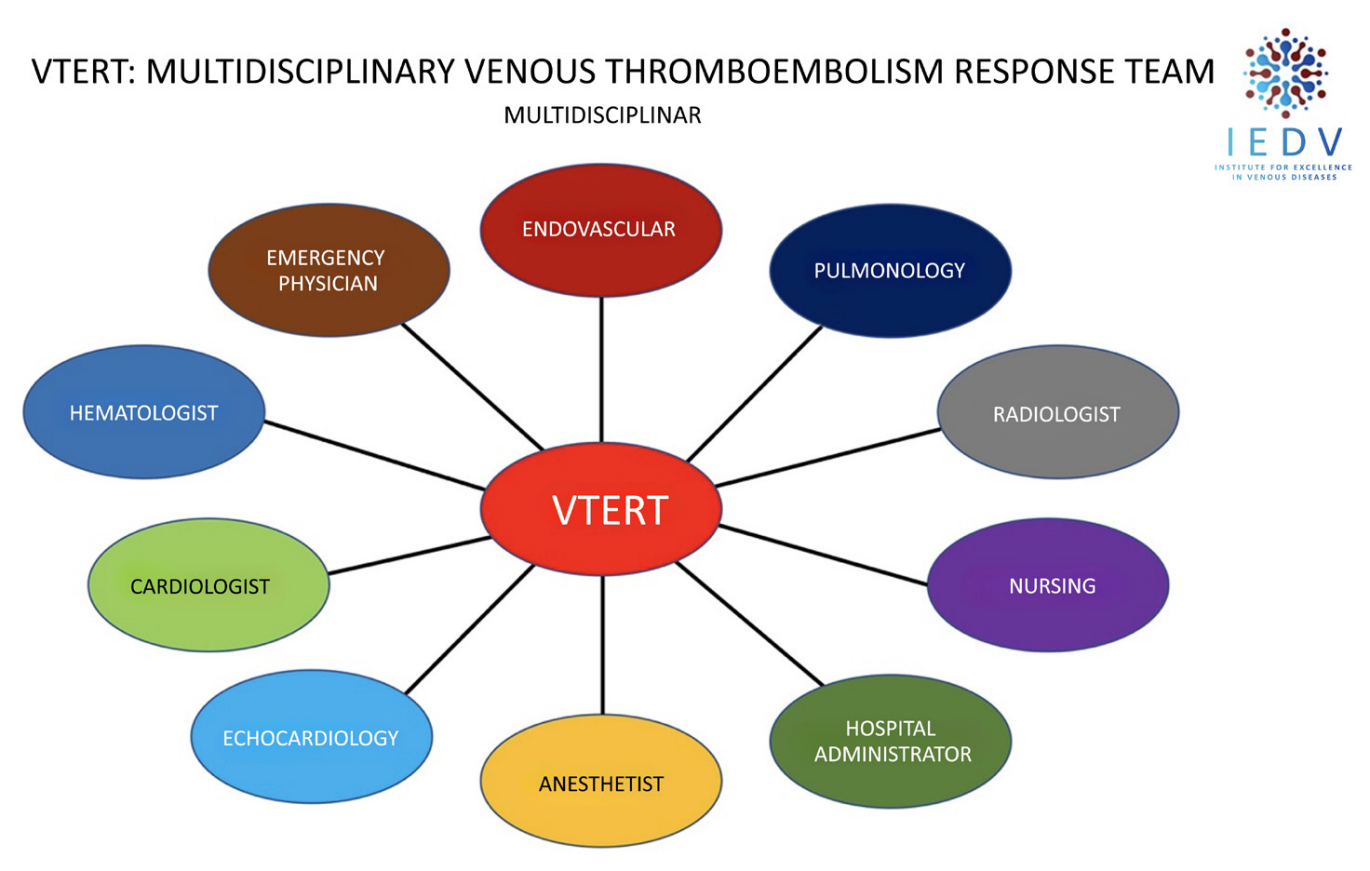
Multidisciplinary concept of care for patients with severe venous thromboembolism centralized by the VTERT® protocol. VTERT = Venous Thromboembolism Response Team; IEDV = Institute for Excellence in Venous Diseases.

The objective of VTERT® is also to optimize use of human resources and materials within the institution, reducing costs over the short, medium, and long terms, while also functioning as a conduit for knowledge diffusion and continuing medical education, agglomerating many different specialties that hitherto worked individually and separately in the same hospital, connecting them along a single common axis of multidisciplinary conduct (Figure 1).

**Figure 2:**
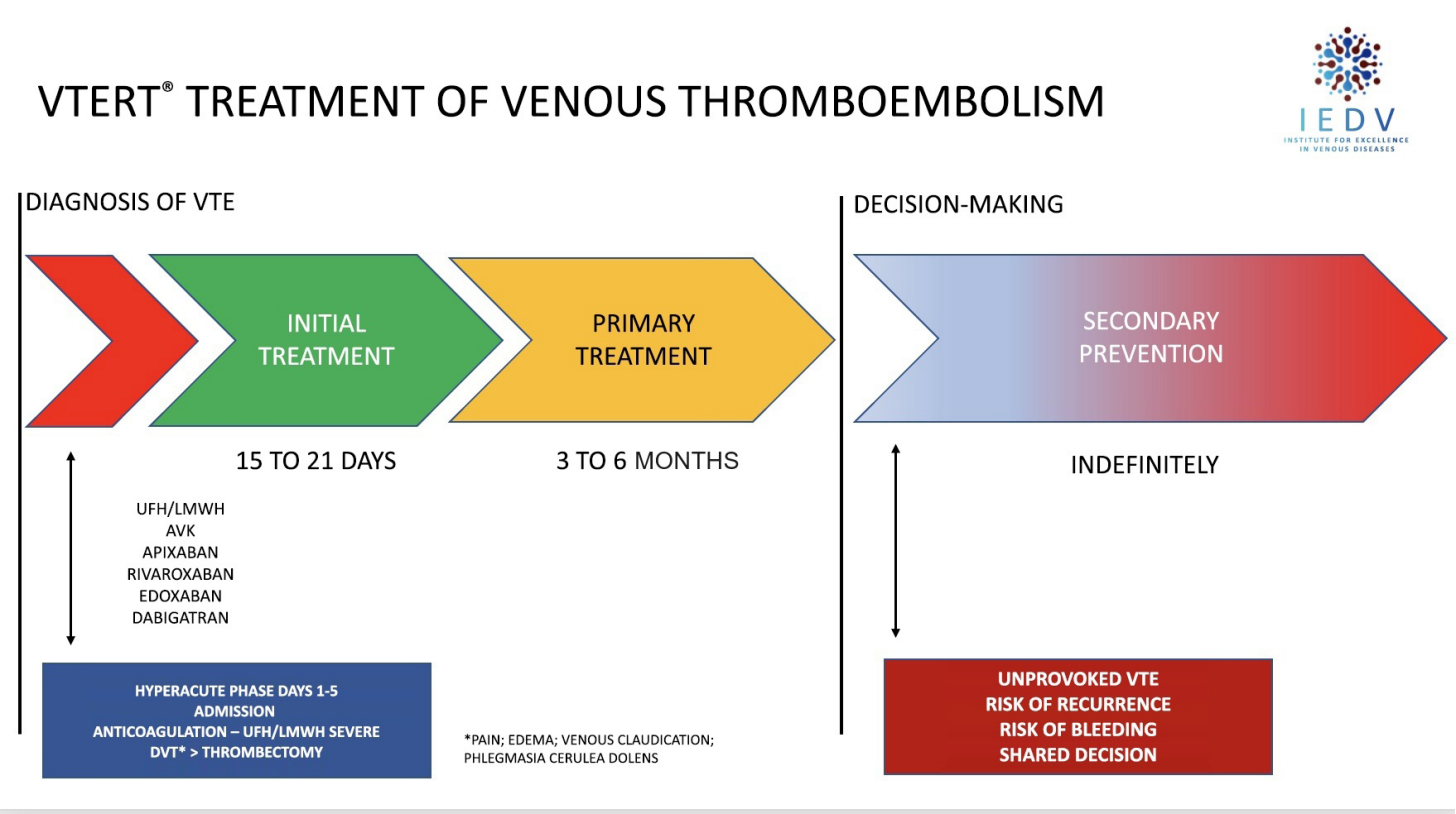
Current treatment for venous thromboembolism plus percutaneous intervention in the hyperacute phase for severe patients following the VTERT® protocol. VTE = venous thromboembolism; AVK = antivitamin K; UFH = unfractionated heparin; LMWH = low molecular weight heparin; VTERT® = Venous Thromboembolism Response Team; IEDV = Institute for Excellence in Venous Diseases.

**Figure 3:**
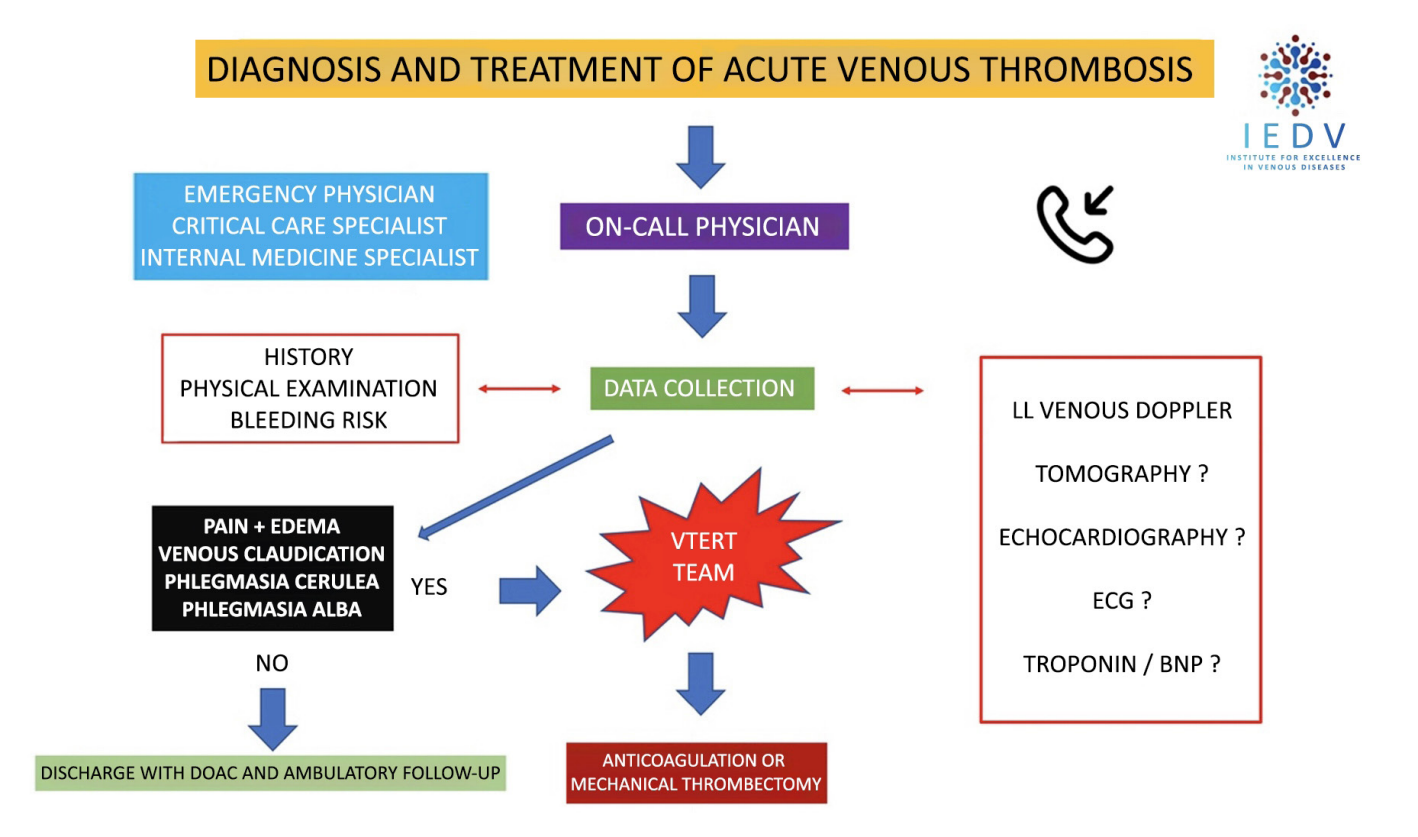
VTERT® protocol for multidisciplinary diagnosis and treatment of severe acute venous thrombosis episodes. VTERT® Team = Venous Thromboembolism Response Team; IEDV = Institute for Excellence in Venous Diseases; DOAC = direct oral anticoagulant; ECG = electrocardiogram; LL = lower limbs; BNP = brain natriuretic peptide.

**Figure 4:**
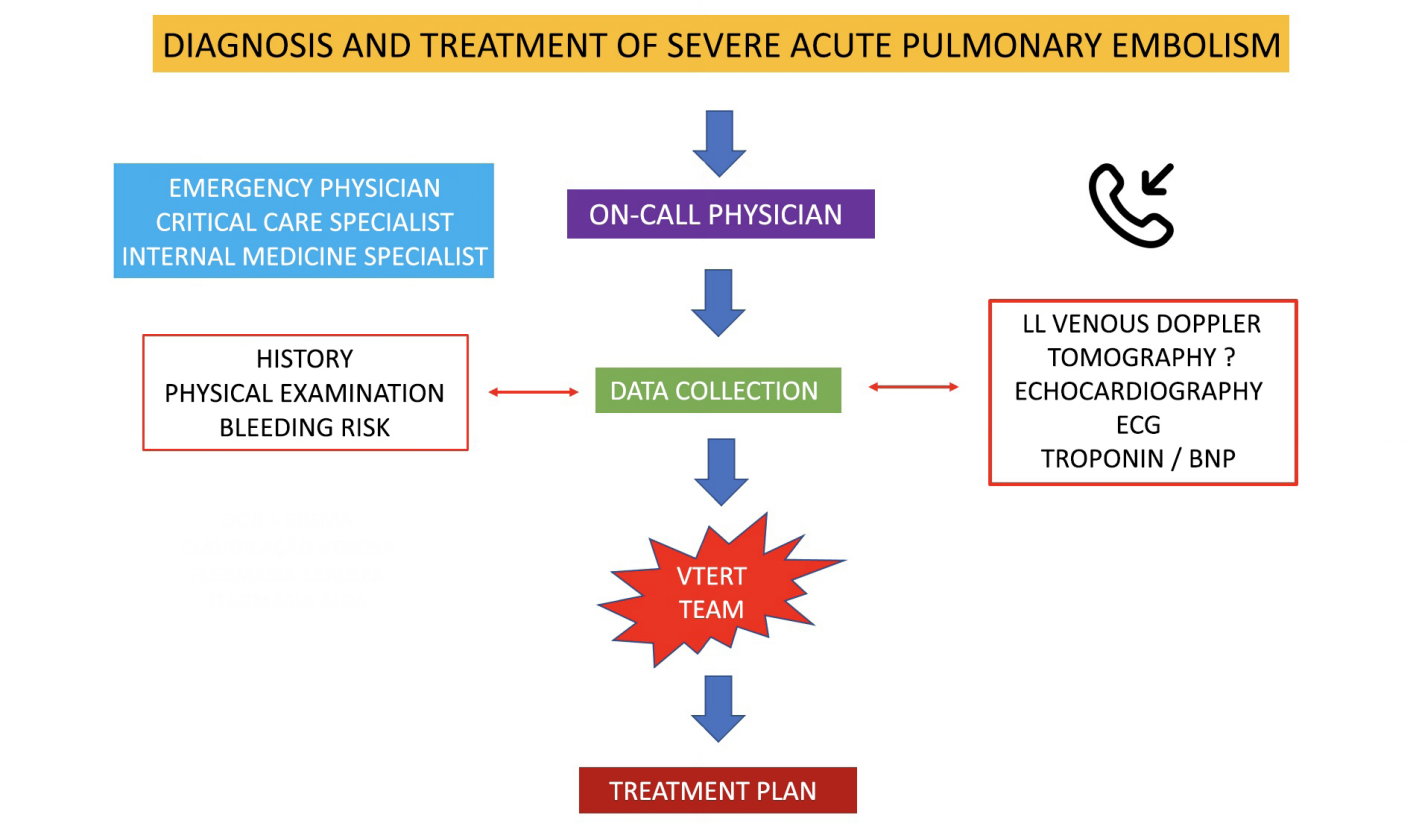
VTERT® protocol for multidisciplinary diagnosis and treatment of severe acute pulmonary embolism episodes. VTERT® = Venous Thromboembolism Response Team; ECG = electrocardiogram; LL = lower limbs; BNP = brain natriuretic peptide.

The basic VTERT® flow diagram incorporates concepts and actions that are already well-established for PE^10,11^ and includes four stages that comprise a continuous and interconnected process of actions:

- **Stage 1**: a patient admitted to the institution is identified as having symptomatic proximal DVT (cava-iliofemoral), with severe clinical status and immediate risk of complications such as PE and/or phlegmasia alba or cerulea dolens, is assessed by the VTERT® team in a conference call via a smartphone or dedicated and exclusive app;
- **Stage 2**: in collaboration with the medical team or the physician responsible for the patient, the VTERT® team discusses the need for supplementary exams [vascular echography with Doppler; troponin; brain natriuretic peptide; echocardiogram; and/or angiotomography with PE and DVT protocol];
- **Stage 3**: the VTERT® team’s on-call physician presents the patient’s clinical and laboratory data via videoconference to the other team members, initiating a multidisciplinary discussion and case review and ending by forming a consensus on treatment;
- **Stage 4**: the established treatment and therapeutic plan are communicated to the interventional physician/team responsible, who immediately communicate with the multidisciplinary team and support manager and carry out the plan (Figures 2, 3, and 4).

The VTERT® team’s primary objectives of immediate VTE treatment are: immediate restoration of venous patency and flow, achieved by removal of the thrombus from the obstructed segment, preventing it from propagating or growing, protecting the patient from PE, and preventing phlebosclerosis, responsible for VTE recurrence and progression to PTS. On restoration of axial venous flow, it is expected that there will be immediate relief from signs and symptoms and normalization of hemodynamic and biochemical functions of regional and systemic venous circulation.

As such, the VTERT® team should preferably be involved in the hyperacute phase of the initial thrombotic event, when the advanced treatment methods available yield the best results (Figures 2, 3,and 4). Pharmacomechanical thrombectomy techniques have been demonstrating safety and efficacy, significantly reducing rates of complications, length of hospital stay, hospital costs, poor outcomes over the medium and long term, and overall mortality.^10-13^

Implementation of the VTERT® concept and the paradigm shift that it enacts are based on scientific evidence, since they follow the same direction as the DVT guidelines published by the European Society for Vascular Surgery (ESVS) in 2021 (Recommendation 34, class IIa evidence level A), which established that early thrombi removal should be considered in selected patients with symptomatic iliofemoral DVT,^10^ and they also follow the guidelines for invasive PE management published by the European Society of Cardiology in 2020.^11^

As has occurred with other rapid response teams, development and application of the VTERT® concept will be exclusively dependent on the interest and involvement of other specialties and the hospitals themselves and on constant development and reassessment, which will enable its propagation and implementation in the greatest possible number of multidisciplinary services, both nationally and internationally. Its clinical and economic effectiveness should be confirmed, initially at centers of excellence, with teams that have been thoroughly trained in the diagnosis and invasive treatment of VTE, following the protocols described above. In this manner, the results of the concept can be fully assessed and its benefits disseminated.
