# Supplementary material for: The paradigm shift in treatment of severe venous thromboembolism
Source: J Vasc Bras. 2024 Mar 4;24:e20230095. doi: 10.1590/1677-5449.202300952 (PMC10939176; doi:10.1590/1677-5449.202300952)
Supplement: Translation

# Translation: A mudança de paradigma no tratamento do tromboembolismo venoso grave

**DOI:** 10.1590/1677-5449.202300951

**Authors:** Fábio Henrique Rossi, Francisco José Osse, Patricia Ellen Thorpe

**Affiliations:** 1 Instituto de Excelência em Doenças Venosas - IEDV, São Paulo, SP, Brasil.

**Keywords:** tromboembolismo venoso, trombose venosa profunda, embolia pulmonar, multidisciplinar, trombectomia, trombólise mecânica

## Abstract

A embolia pulmonar (EP) é a terceira maior causa de morte cardiovascular e a principal de morte evitável intra-hospitalar no mundo. O conceito PERT® (do inglês, *pulmonary embolism response team*) envolve seu diagnóstico e tratamento precoce e multidisciplinar. A trombose venosa profunda (TVP) é a sua causa inicial na maioria dos casos e é responsável por complicações como a recidiva tromboembólica, a síndrome pós-trombótica e a hipertensão pulmonar tromboembólica crônica. Uma abordagem inicial semelhante ao PERT nos casos de TVP ilíaco-femoral grave pode reduzir não apenas o risco imediato de EP e morte, mas também suas sequelas tardias. Novas técnicas percutâneas e aparatos de trombectomia mecânica para o tromboembolismo venoso (TEV) vêm demonstrando resultados clínicos encorajadores. Propomos o desenvolvimento de um conceito ampliado de resposta rápida ao TEV, que envolve não apenas a EP (PERT®) mas também os casos graves de TVP: o time de resposta rápida para o TEV (TRETEV®), ou do inglês *Venous Thromboembolism Response Team* (VTERT®).

## INTRODUÇÃO

A doença venosa crônica (DVC) é a enfermidade vascular de maior prevalência, podendo provocar severo comprometimento da qualidade de vida, necessidade de cuidados médicos de longa duração, e custo financeiro pessoal e social elevado. O tromboembolismo venoso (TEV) está relacionado à trombose venosa profunda (TVP), e/ou à embolia pulmonar (EP) e às síndromes obstrutivas venosas, que podem trazer riscos imediatos e sequelas tardias, sendo a mais prevalente a síndrome pós-trombótica (SPT), que acomete entre 30% e 50% dos indivíduos diagnosticados com TVP proximal ilíaco-femoral^1^ . Apesar de todo o desenvolvimento de fármacos anticoagulantes cada vez mais seguros e eficazes, os índices de complicações tromboembólicas crônicas têm permanecido os mesmos, demonstrando claramente haver espaço para a busca de melhora na conduta terapêutica.

A EP representa a terceira causa mais comum de óbito cardiovascular, ficando atrás apenas do infarto agudo do miocárdio (IAM) e do acidente vascular cerebral (AVC), e é a principal causa evitável de morte intra-hospitalar^1^ . As síndromes obstrutivas abomino-pélvicas (síndrome de May-Thurner-Cockett, síndrome de Nutcracker e doença venosa pélvica) hoje estão associadas à ocorrência de TEV agudo e à DVC avançada, além disso, podem provocar sinais e sintomas incapacitantes, como dor lombar e hematúria, dor pélvica crônica, dispareunia e varicocele. Muito embora as TVPs acometam preferencialmente os membros inferiores, o uso crescente de cateteres venosos centrais (PICC, do inglês *peripheral inserted central cateter*, marcapasso, hemodiálise etc.) vem aumentando a incidência da TVP e da obstrução venosa central, associado à diminuição da sobrevida em pacientes renais crônicos hemodialíticos^2^ .

A incidência de TEV na população geral varia de 60 a 165 novos casos por 100.000 pessoas por ano, sendo a TVP a apresentação mais comum (85% do total de casos). Quando se analisa a incidência de TVP, de EP ou os dos dois eventos combinados em relação à faixa etária, observa-se um aumento exponencial a partir da quinta/sexta década de vida, e outros fatores de risco frequentes na população ocidental, como obesidade, sedentarismo, tabagismo, e terapia hormonal, podem aumentar sua incidência^3^ .

A TVP é classicamente dividida em duas fases: a aguda, na qual o trombo apresenta a composição histológica de coágulo, com múltiplas fibras de fibrina encarcerando hemácias, plaquetas e plasma, o que também promove a adesão do coágulo à parede venosa; e a fase tardia, quando as reações intrínsecas e extrínsecas, em resposta à formação e presença do trombo, agem para a sua dissolução, mas ocorre também a organização fibrótica (flebosclerose) que, por sua vez, é responsável pela obstrução e represamento do fluxo venoso e, em última instância, pela SPT. Na fase aguda, pode haver descolamento de fragmentos de coágulos para a circulação pulmonar, desencadeando a mais temida das suas complicações, a EP, que a depender do volume do coágulo, poder levar ao óbito. Nos sobreviventes, as sequelas obstrutivas, provocadas pela fleboesclerose, que ocorrem na fase crônica, podem provocar obstruções venosas abdominopélvicas, que por sua vez, são fatores de risco para novos eventos agudos e recidiva da EP^2,3^ .

Dados de uma grande companhia de seguros dos EUA revelaram que ocorrem 5,2 reivindicações por 1.000 segurados e indicaram que a DVC grave é responsável por metade delas. Verificou-se que 8,6% da população dos EUA (idade média 46,3 anos) têm uma doença venosa clinicamente relevante e que, em 3% desses indivíduos, o problema é grave, exigindo tratamento médico, ou internação hospitalar^3^ . A úlcera varicosa acomete anualmente, em média, 1,5 a 2 milhões de indivíduos nos EUA, a um custo anual médio de U$ 850, sem que estejam incluídos nesse valor os custos hospitalares, dias de trabalho perdidos e produtos médicos^3^ . Assim, reconhecer que os distúrbios venosos são verdadeiramente uma questão de saúde pública é o primeiro passo para o desenvolvimento de uma abordagem responsável, através da qual se possa efetivamente intervir antes que a doença venosa se torne crônica, incapacitante e dispendiosa^4^ .

Sabemos que a EP é uma doença grave e associada a alto risco de mortalidade que, por sua vez, pode ser reduzida, caso o seu diagnóstico e tratamento sejam feitos precocemente. Entretanto, o diagnóstico nem sempre é considerado na ocorrência de dor aguda torácica, colapso cardiopulmonar e instabilidade hemodinâmica. Não é incomum que o paciente acometido, assistido pelo médico emergencista, ou intensivista, que são os responsáveis pelo diagnóstico, e pela decisão terapêutica inicial, não considere a EP no diagnóstico diferencial das síndromes cardiopulmonares agudas. O especialista intervencionista, na maioria das vezes, é chamado apenas tardiamente, nos casos já mais graves, muitas vezes já em tratamento por infusão sistêmica de agentes trombolíticos, apresentando hemorragia, instabilidade hemodinâmica e choque cardiogênico, situações clínicas de difícil reversão. O desenvolvimento de técnicas percutâneas de tratamento, sobretudo de trombectomia mecânica, os seus resultados clínicos encorajadores atuais, mas também as diferenças no nível de experiência, e curva de aprendizado das equipes intervencionistas, provocam a heterogeneidade, e um aumento na complexidade da tomada de decisão terapêutica, e na condução dos casos de EP grave. Isso vem estimulando o desenvolvimento de times multidisciplinares de resposta rápida para a EP (PERT®, do inglês *pulmonary embolism response team*), os quais incluem profissionais de diversas especialidades, que se envolvem simultaneamente no processo de diagnóstico, avaliação e tratamento, principalmente naqueles pacientes identificados como sendo de alto risco de morte^5^ .

O conceito do PERT® inclui o aprimoramento no atendimento, e na condução terapêutica dos casos de EP, com o objetivo básico de prevenir morte evitável, por complicações cardiopulmonares, promovendo o início imediato do tratamento, através de um consenso obtido entre diferentes especialidades, após a avaliação individualizada de casos identificados dentro do hospital. Visa melhorar a organização e utilização dos recursos da instituição, com cada membro e especialidade contribuindo com seu conhecimento específico para formatar e padronizar as condutas nos casos de EP grave. Como consequência dessas ações, observa-se um aumento gradual, mas sustentado, da difusão dos conhecimentos gerais e específicos no uso de terapias avançadas, integrando essas diversas especialidades em um mesmo objetivo, determinando não apenas maior eficácia e segurança, como também redução de custos^6^ . O conceito e o fluxograma pioneiros do PERT® foram instituídos no Hospital Geral de Massachussetts (Harvard Medical School), e a revisão dos resultados de diversos serviços PERT® criados vem demonstrando redução na morbimortalidade, no tempo médio de internação, e nos custos gerais do tratamento^7-9^ . Se reconhecemos a necessidade clínica e os promissores resultados obtidos pela “intervenção imediata e antecipada para evitar complicações” em pacientes portadores de EP com instabilidade hemodinâmica, além das indicações de intervenção em casos graves de TVP aguda proximal, parece razoável, como cirurgiões vasculares, e especialistas no tratamento invasivo de TEV, a aplicação dos mesmos conceitos do PERT® não apenas nos portadores de EP, mas também nos casos de TVP grave. Dessa forma, estaremos não somente aliviando os sintomas agudos, como também prevenindo a EP, sua recidiva, e complicações crônicas^10^ .

Considerando esses conceitos, acreditamos que existe a urgente necessidade de uma abordagem diagnóstica e terapêutica multidisciplinar para os casos graves de TVP, e propomos uma mudança no paradigma da abordagem clínica nos casos graves, que acometem o eixo venoso ilíaco-femoral, tal qual vem ocorrendo na EP. Obtivemos dispensa da aprovação no comitê de ética em pesquisa pela nossa instituição.

## CONCEITO VTE + PERT® = VTERT®

O conceito VTERT® baseia-se em uma abordagem multidisciplinar não apenas para os casos de EP, mas também para aqueles casos de TVP proximal grave, em que existe maior risco de SPT e EP, em pacientes internados nos diversos serviços de uma mesma instituição hospitalar. O objetivo principal é acelerar a rapidez na identificação, na avaliação, no diagnóstico e no tratamento desses pacientes através de um processo preestabelecido de estratificação de risco, atuando nos mesmos moldes dos times clínicos de resposta rápida (IAM, AVC e EP) na fase aguda, já amplamente instituídos em diversos hospitais de diversos países^11^ .

A resposta imediata aos pedidos de avaliação desses casos é seguida de discussão por equipe multidisciplinar, preestabelecida, para a correta decisão sobre o tratamento a ser instituído, visando não somente o resultado clínico imediato, com melhora dos sintomas e redução de risco imediato de morte, como também a prevenção de complicações, e recidivas futuras.

O objetivo do VTERT® também é otimizar o uso de recursos humanos e materiais dentro da instituição, reduzindo os custos envolvidos a curto, médio e longo prazo, funcionando também como difusor de conhecimento e educação médica continuada, aglutinando diversas especialidades, que ora trabalham de forma individualizada dentro de um mesmo ambiente hospitalar, conectando-as num mesmo eixo comum de conduta multidisciplinar (Figura 1).

**Figura 1:** Conceito multidisciplinar de atendimento ao paciente portador de tromboembolismo venoso grave centralizado pelo protocolo VTERT®. VTERT = *Venous Thromboembolism Response Team*; IEDV = Instituto de Excelência em Doenças Venosas.

O fluxograma básico do VTERT®, além de considerar os conceitos e ações já bem estabelecidos para a EP^10,11^ , possui quatro etapas num processo contínuo e interligado de ações:

- **Etapa 1**: o paciente admitido e internado na instituição, identificado como sendo portador de TVP proximal (cava-ilíaco-femoral) sintomática, com quadro clínico grave, e risco imediato de complicações como EP e/ou *phlegmasia alba* ou *cerúlea dolens*, é avaliado pela equipe VTERT® via chamada de conferência realizada por celular e/ou aplicativo dedicado e de uso exclusivo;
- **Etapa 2**: a equipe VTERT®, em colaboração com a equipe médica ou com o médico responsável pelo paciente, discute a necessidade de realização de exames complementares [ecografia com Doppler vascular; troponina; peptídeo natriurético cerebral (BNP); ecocardiograma; angiotomografia com protocolo para EP e TVP];
- **Etapa 3**: o médico plantonista da equipe VTERT® apresenta os dados clínicos e laboratoriais do paciente via videoconferência para os demais integrantes do grupo, procedendo discussão multidisciplinar e revisão do caso e finalizando com a formulação de um consenso de tratamento;
- **Etapa 4**: o tratamento e plano terapêutico estabelecidos são comunicados ao médico/equipe intervencionista responsável, que imediatamente comunica a equipe multidisciplinar e gestora de suporte e executa a realização do mesmo (Figuras 2, 3 e 4).

**Figura 2:** Tratamento atual do tromboembolismo venoso acrescido da intervenção percutânea na fase hiperaguda em pacientes graves segundo o protocolo VTERT®. TEV = tromboembolismo venoso; AVK = antivitamina K; HNF = heparina não fracionada; HBPM = heparina de baixo peso molecular; TVP = trombose venosa profunda; VTERT® = *Venous Thromboembolism Response Team*; IEDV = Instituto de Excelência em Doenças Venosas.

**Figura 3:** Protocolo VTERT® de diagnóstico e tratamento multidisciplinar para episódios agudos e graves de trombose venosa aguda. VTERT® Team = *Venous Thromboembolism Response Team*; IEDV = Instituto de Excelência em Doenças Venosas; DOAC: direct oral anticoagulants; ECG = eletrocardiograma; MMII = membros inferiores; BNP = brain natriuretic protein.

**Figura 4:** Protocolo VTERT® de diagnóstico e tratamento multidisciplinar para episódios agudos e graves de embolia pulmonar. VTERT® Team = *Venous Thromboembolism Response Team*; ECG = eletrocardiograma; MMII = membros inferiores; BNP = brain natriuretic protein.

Os principais objetivos do tratamento imediato do TEV pelo time VTERT® são: restituição imediata da perviedade e do fluxo venoso, obtida pela remoção do trombo presente no segmento obstruído, impedindo sua propagação e extensão, protegendo o paciente contra EP e prevenindo a flebosclerose, responsável pela recorrência do TEV e evolução para SPT. Ao restaurar o fluxo venoso axial, espera-se que ocorram de imediato o alívio de sinais e sintomas e a normalização das funções hemodinâmicas e bioquímicas da circulação venosa regional e sistêmica.

Nesse sentido, a atuação do VTERT® deve ocorrer, preferencialmente, na fase hiperaguda do evento trombótico inicial, quando os métodos avançados de tratamento disponíveis apresentam os melhores resultados (Figuras 2, 3 e 4). As técnicas de trombectomia farmacomecânica vêm demonstrando segurança e eficácia, reduzindo significativamente os índices de complicações, tempo de internação, custos hospitalares, evoluções desfavoráveis a médio e longo prazo e a mortalidade geral^10-13^ .

A implementação do conceito VTERT® e a mudança de paradigma que ele traz possuem embasamento científico, pois seguem na mesma direção das orientações para o manuseio da TVP, publicadas pela Sociedade Europeia de Cirurgia Vascular (ESVS) em 2021 (Recomendação 34, classe IIa nível A de evidência), que estabelecem que, em pacientes selecionados e portadores de TVP iliofemoral sintomática, a remoção precoce dos trombos deve ser considerada^10^ , além de também seguirem as orientações para o manuseio invasivo da EP, publicada pela Sociedade Europeia de Cardiologia em 2020^11^ .

Assim como ocorreu com os outros “times” de resposta rápida, o desenvolvimento e a aplicação do conceito VTERT® dependerão exclusivamente do interesse e envolvimento das demais especialidades, dos hospitais e da evolução e reavaliação constante, que permitam sua propagação e aplicação no maior número possível de serviços multidisciplinares, nacionais e internacionais. Sua efetividade clínica e econômica deve ser verificada, inicialmente, em centros de excelência, com equipes bem treinadas, no diagnóstico e tratamento invasivo do TEV, seguindo os protocolos acima descritos. Desse modo, os resultados poderão ser bem avaliados, e os benefícios, difundidos.
